# Congenital short bowel syndrome: Clinical aspects by systematic review

**DOI:** 10.1002/jpr3.70221

**Published:** 2026-08-13

**Authors:** Barblin Remund, Susanne Schibli, Christiane Sokollik

**Affiliations:** ^1^ Department of Paediatrics, Inselspital, Division of Paediatric Gastroenterology, Hepatology and Nutrition Bern University Hospital, University of Bern Bern Switzerland

**Keywords:** congenital diarrhea, genetic testing, parenteral nutrition, radiological imaging, short gut

## Abstract

**Objectives:**

Congenital short bowel syndrome (CSBS) is a rare intestinal disorder characterized by inborn shortening of the bowel with mainly mutations in Coxsackie and Adenovirus receptor‐like membrane protein (CLMP) and Filamin A (FLNA) genes. Clinical features are not well described; therefore, we aim to improve patient care by evaluating diagnostic approaches and identifying prognostic factors through the analysis of published cases.

**Methods:**

We performed a systematic review of cases published between 2000 and 2024.

**Results:**

Genetic analysis in 35 of the 61 CSBS cases revealed a CLMP mutation in 57.1%, FLNA in 25.7%, and others in 17.1%. Consanguinity was common with 42.4%. Nine families had affected siblings. Most cases were term births with normal birth weight. The first symptoms occurred after a median of 0.6 weeks, mostly vomiting and diarrhea. Median (range) small bowel length was 50.0 (20–85) cm. Comorbidities included malrotation in 87.2%. Nearly all cases required parenteral nutrition (PN), with 60% achieving enteral autonomy after a median follow‐up of 15 months. Nine children died before the age of 2 years, six due to sepsis. No intestinal malignancy was reported.

**Conclusions:**

CSBS presents shortly after birth with the majority of affected cases achieving enteral autonomy in infancy, which may lead to an underestimation of incidence rates. Radiological imaging can be used to determine bowel length for diagnostic purposes and to initiate genetic counseling. Genetic testing is essential for mutation‐specific prognosis and patient management. Complications associated with PN pose the greatest risk for adverse outcomes.

## Abstract (highlights)

**What is Known:**

- Congenital short bowel syndrome (CSBS) is a rare intestinal disorder with inborn shortening of the bowel and frequently associated with malrotation.
- Patients with CSBS present with different genetic mutations.
- Prognosis is primarily affected by infections related to parenteral nutrition.

**What is New:**

- Prioritizing radiological imaging and genetic testing as the first line of diagnostic work‐up may help to avoid diagnostic laparotomies.
- Filamin A mutations result in a more severe phenotype with a higher prevalence of associated malformations compared to Coxsackie and Adenovirus receptor‐like membrane protein mutations.
- Prognosis is favorable with achievement of enteral autonomy in 60% of cases within 15 months.

## 1 INTRODUCTION

Congenital short bowel syndrome (CSBS) was first described in 1969.^1^ CSBS is a rare intestinal disorder, which is characterized by an inborn shortening of the small bowel often combined with a malrotation of the gut. This is in divergence to (1) secondary short bowel syndrome (SBS) due to surgical shortening in cases of gastroschisis, necrotizing enterocolitis, intestinal atresia, or Hirschsprung disease, (2) functional SBS where the length is intact, and (3) absence of the midgut where a vascular insult during embryonic life is suggested.

The two most common mutations causing CSBS are in the Coxsackie and Adenovirus receptor‐like membrane protein (CLMP) gene with an autosomal recessive inheritance^2^ and in the Filamin A (FLNA) gene with an X‐linked inheritance.^3^ While CLMP encodes a transmembrane protein critically involved in intestinal development, FLNA encodes a cytoskeletal protein, and its precise role in intestinal development remains to be determined.^4^, ^5^ Furthermore, mutations in the Dynein Cytoplasmic 2 Light Intermediate Chain 1 (DYNC2LI1) gene,^6^ the H.20‐like homeobox 1 (HLX) gene,^7^ the Forkhead box F1 (FOXF1) gene^8^ and duplication of chromosome 16q24.1 involving FOXF1^9^, ^10^, ^11^ have been detected in patients with CSBS. Patients present with chronic diarrhea, vomiting, and failure to thrive.^12^ They may require long‐term parenteral nutrition due to intestinal failure and malabsorption. Because CSBS is very rare and its presenting symptoms are common to other pathologies, the diagnosis is often delayed and difficult to make.

We conducted a systematic review and extracted published patients’ data to analyze clinical characteristics and outcomes in conjunction with the presentation of a new case. We aim to improve patient care by suggesting diagnostic approaches and deriving information about prognostic factors.

## 2 METHODS

### 2.1 Ethics statement

Caregivers of our patient provided written informed consent.

### 2.2 Search strategy and selection criteria

For this systematic review, a search in PubMed was performed on 19.07.2024, using the search terms “congenital short bowel,” “congenital short bowel syndrome,” “congenital short small bowel,” “congenital short gut,” “congenital short gut syndrome,” “congenital short intestine,” and “congenital short small intestine.” The search terms were combined using the Boolean operator OR to identify the most relevant publications. The search was limited to the English language and to publications from 2000 to 2024. Inclusion criteria was congenital short bowel diagnosis. Exclusion criteria were case presentations of animals, publications analyzing previously published cases and non‐CSBSs (e.g., after bowel resection). Abstracts and if unclear full texts from the search results were screened and reviewed for relevance and the previously mentioned inclusion criteria were applied for selection. Additionally, reference lists of the relevant publications were searched to identify further relevant publications. Any discrepancies in decision‐making between the reviewers were resolved through discussion.

### 2.3 Data extraction and assessment

The following patients data were extracted: sex, gestational age, consanguinity of parents, age at first symptoms, type of first symptoms, diagnostic approaches, small bowel length, malrotation of the gut, requirement of parenteral nutrition, duration of parenteral nutrition, achievement of enteral autonomy, comorbidities in general and specifically comorbidities of the gastrointestinal tract, complications during follow‐up, and underlying genetic mutations. Variables not reported in a specific case were labeled as not available (N/A). The extracted data was recorded in a Microsoft Excel database. Cases were divided into four subgroups based on their genetic background: cases with CLMP mutations, cases with FLNA mutations, cases with other genetic backgrounds and cases without genetic testing mentioned. For cases that were reported in more than one publication, available data were combined.

### 2.4 Statistical analysis

Descriptive statistics were used depending on the type of data, including numbers (percentages), medians (interquartile ranges [IQR], or range), and means (standard deviations [SD]).

## 3 RESULTS

Our search strategy identified 45 publications. One was excluded during abstract screening due to being an animal study, and 44 were assessed based on full text, of which 16 were excluded (Figure S1) Screening of the reference list of the included publications led to the inclusion of four additional publications. In total, we included 32 publications and extracted data of 61 cases with CSBS.^2^, ^6^, ^7^, ^8^, ^9^, ^13^, ^14^, ^15^, ^16^, ^17^, ^18^, ^19^, ^20^, ^21^, ^22^, ^23^, ^24^, ^25^, ^26^, ^27^, ^28^, ^29^, ^30^, ^31^, ^32^, ^33^, ^34^, ^35^, ^36^, ^37^, ^38^, ^39^

Patients’ characteristics are summarized in Table 1. Genetic testing was reported in 35/61 cases with a CLMP mutation found in the majority. All nine FLNA cases were male, explained by its X‐linked inheritance. Consanguinity was common (42.4%) with the highest rate in CLMP (53.9%). Prenatal examinations were reported for 12 cases, with intestinal abnormalities detected in three cases. Nearly all cases were term with a normal birth weight (median [IQR] *z*‐score −0.6 [1.76]). Median [IQR] age at first onset of symptoms was 0.6 [2.1] weeks. The leading symptoms at first presentation were vomiting (48.7%), bilious vomiting (29.7%), and diarrhea (21.6%). CSBS was diagnosed during laparotomy in 35 cases, autopsy in three cases, imaging in three cases, and laparoscopy in one case. Median [range] small bowel length in under 4‐month‐old was 50.0 [20–85] cm, with a slightly longer small bowel length of 68.0 [45–75] cm in FLNA cases and cases with other mutations (65.0 [45–85] cm). A malrotation was found in 43 cases. Comorbidities were reported in 37 cases, with the most common being gastrointestinal, followed by dysmorphic features and neurological abnormalities. Nearly all cases needed parenteral nutrition (PN). Achievement of enteral autonomy was reported in 24/40 (60%) cases with a median [IQR] follow‐up time of 15 [21.5] months. When comparing small bowel length with the achievement of enteral autonomy, cases that achieved enteral autonomy had a longer median small bowel length; however, these cases also had a longer follow‐up time (Table S1). Duration of PN requirement before achieving enteral autonomy was mentioned in 10 cases with a median [IQR] of 4.5 [12.5] months. Medical complications during follow‐up were described in 24 cases. Nine cases died during follow‐up, which was mainly due to sepsis (six cases) and occurred in cases <2 years of age. No intestinal malignancy was reported.

**Table 1:** Characteristics of published cases.

	All	CLMP	FLNA	Other mutations	No genetics performed	Comments
*N* (%)	61	20 (32.8)	9 (14.8)	6 (9.8)	26 (42.6)	Other mutations: DYNC2LI1 gene mutation in 1, HLX gene mutation 1, FOXF1 gene mutation 1, duplication of chromosome 16q24.1 involving FOXF1 3
Gender, male, *n* (%)	32 (52.5)	8 (40)	9 (100)	2 (33.3)	13 (50)	
Gender, female, *n* (%)	29 (47.5)	12 (60)	0 (0)	4 (66.7)	13 (50)	
No. mentioned consanguinity, *n*	33	13	4	3	13	
Consanguinity, yes, *n* (%)	14 (42.4)	7 (53.9)	0 (0)	1 (33.3)	6 (46.2)	
No. mentioned prenatal examinations, *n*	12	2	3	4	3	High suspicious for intestinal malrotation in 1, persistent bowel dilation 1, prominent gastric bubble 1
No. mentioned gestational age, *n*	37	11	8	3	15	
Gestational age, weeks, mean [SD]	38.1 [1.9]	38.1 [1.8]	38.9 [0.6]	37.0 [2.0]	37.9 [2.3]	
No. mentioned birthweight, *n*	34	11	6	3	14	
Birthweight, grams, mean [SD]	2814 [439]	2841 [544]	3049 [359]	2597 [435]	2795 [550]	
Birthweight, *z*‐score, median [IQR]	−0.569 [1.764]	−0.699 [2.291]	−0.698 [0.855]	−0.414 [‐]	−0.315 [1.751]	
No. mentioned age at first symptoms, *n*	38	9	8	3	18	
Age at first symptoms, weeks, median [IQR]	0.64 [2.1]	0.57 [1.13]	1.5 [60.68]	0.71 [1.5]	0.5 [1.75]	
No. mentioned first symptoms, *n*	37	11	6	2	18	vomiting in 18, bilious vomiting 11, diarrhea 8, abdominal distention 10, failure to thrive 3, abdominal pain 2, weight loss 2, jaundice 2, frequent defecation 2, constipation 1, bloody stools 1, hematemesis 1, gastrooesophageal reflux 1, flatulence 1
No. mentioned diagnostic method, *n*	42	11	7	4	20	
Laparotomy/laparoscopy, *n*	36	10	3	4	19	
Imaging, *n*	3	0	2	0	1	Upper gastrointestinal tract series in 3
No. mentioned small bowel length, *n*	41	16	3	3	19	in <4 months olds
Small bowel length, cm, median [range]	50.0 [20–85]	48.5 [26–76]	68.0 [45–75]	65.0 [45–85]	50.0 [20–80]	
No. mentioned malrotation, *n*	47	14	7	5	21	
Complete malrotation, *n* (%)	41 (87.2)	13 (92.9)	6 (85.7)	4 (80)	18 (85.7)	
Incomplete malrotation, *n* (%)	2 (4.3)	0 (0)	0 (0)	1 (20)	1 (4.8)	
No malrotation, *n* (%)	4 (8.5)	1 (7.1)	1 (14.3)	0 (0)	2 (9.5)	
No. mentioned comorbidities GIT, *n*	29	8	6	3	12	Intestinal pseudoobstruction in 8, affected colon 6, intestinal neuronal dysplasia 5, intestinal atresia 3, pyloric stenosis 3, omphalocele 2, intestinal dysmobility 2, pyloric hypertrophy 2, stenosis duodenum 1, pyloric atresia 1, gastric heterotopia 1, no ileocecal valve 1, anular pancreas 1, mesenterium commune 1, aplasia cecum and appendix 1
No. mentioned other comorbidities, *n*	19	3	7	6	3	Dysmorphic signs in 10, diverse 10, neurological 7, cardiovascular 6, uretrovesical 3, renal 3
No. mentioned duration follow‐up, *n*	35	11	5	2	17	
Duration follow‐up, months, median [IQR]	15 [21.5]	17 [11]	34 [191]	31.5 [7.5]	12 [19.5]	
No. mentioned enteral autonomy, *n*	40	11	7	3	19	
Achieved enteral autonomy, *n* (%)	24 (60)	9 (81.8)	4 (57.1)	1 (33.3)	10 (52.6)	
PN, *n*	38 (95)	11 (100)	5 (71.4)	3 (100)	19 (100)	
No. mentioned PN duration, *n*	10	5	0	1	4	
PN duration, months, median [IQR]	4.5 [12.5]	4 [19.7]	‐	15 [‐]	4 [4.3]	
No. mentioned complications, *n*	24	4	5	4	11	Death in 9, sepsis 8, volvulus 5, intestinal obstruction 2, D‐lactate acidosis 1, milk protein intolerance 1, cholelithiasis 1, cholestatic liver disease with fibrosis 1, lactobezoar at terminal ileum 1, meconium peritonitis 1, gastrooesophageal reflux 1, catheter infection 1, necrotizing enterocolitis 2, upper gastrointestinal hemorrhage 1, liver failure 1

*Note*: More than one first symptom, comorbidity, or complication may be recorded per case.

Abbreviations: CLMP, Coxsackie and Adenovirus receptor‐like membrane protein; DYNC2LI1, dynein cytoplasmic 2 light intermediate chain 1; FLNA, Filamin A; FOXF1, Forkhead box F1; GIT, gastrointestinal tract; HLX, H.20‐like homeobox 1; IQR, interquartile range; PN, parenteral nutrition; SD, standard deviation.

**Figure 1:**
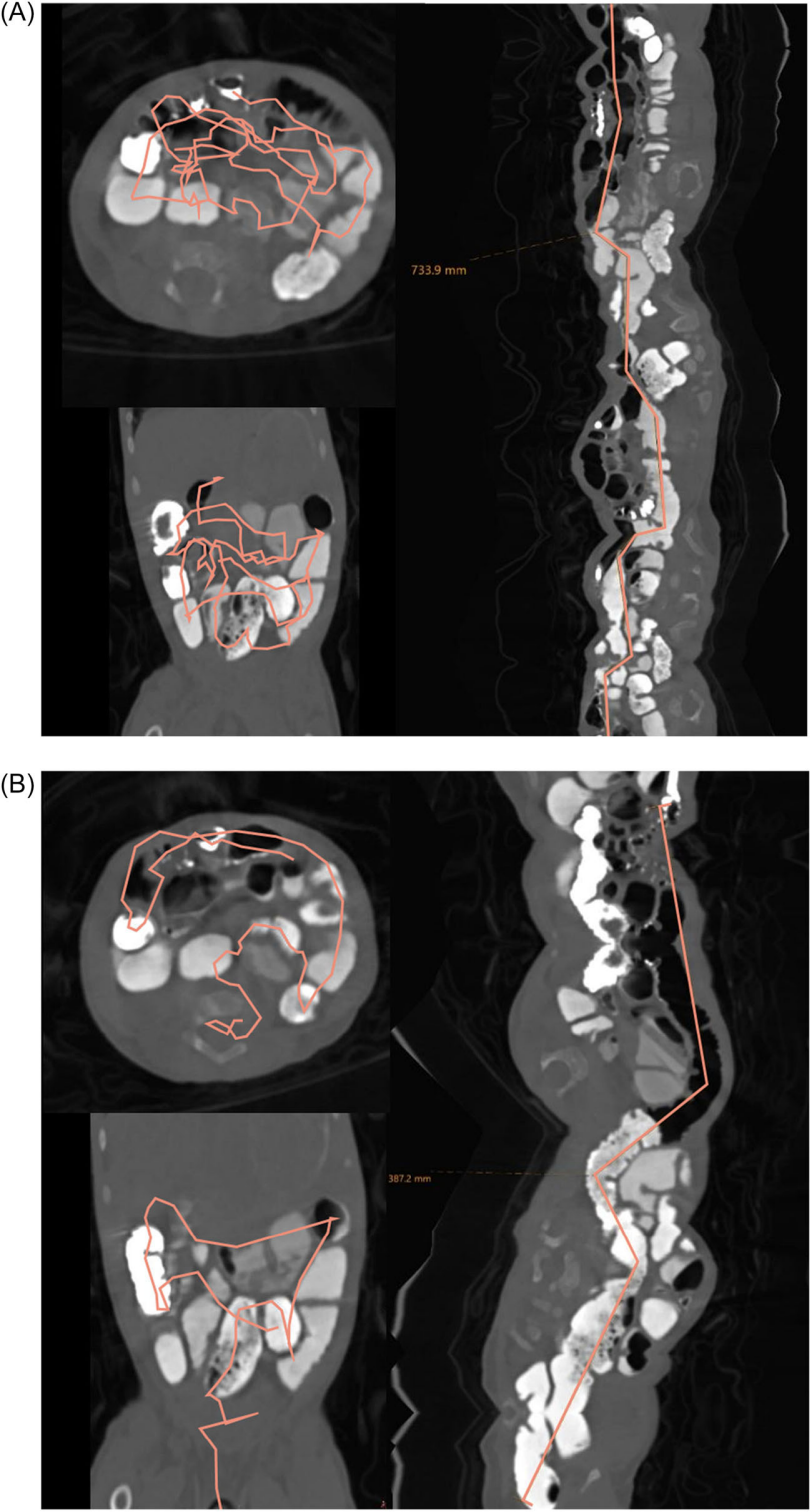
(A) Contrast enhanced computer tomography of the abdomen of our patient to determine small bowel length. Red line indicates measurement. Total length 73.39 cm. (B) Contrast enhanced computer tomography of the abdomen of our patient to determine colon length. Red line indicates measurement. Total length 38.72 cm.

An additional female case at our institution presented at 3 weeks of age with hypovolemic shock, resulting from progressive diarrhea and vomiting since birth. Initially, food protein‐induced enterocolitis syndrome was suggested with all work‐up being inconclusive (Table S2). An endoscopy with biopsies showed no structural changes or signs of inflammation. Computer tomography established a small bowel length of 73.4 cm (normal range for age: 239 cm, SD 18 cm) and a colon length of 38.7 cm (expected normal for age: 57 cm, SD 3 cm)^40^ (Figure 1A,B). Trio‐exome sequencing confirmed a CLMP missense variant (c.328 A > C p.(Thr110Pro)) in exon 3 and a deletion in exon 1, leading to a diagnosis of CSBS. PN could be stopped at the age of 8 months with good weight gain.

## 4 DISCUSSION

Our analysis of 61 CSBS cases published since 2000 and our own case showed that enteral autonomy can be achieved in most cases with CBSC in infancy. The optimal small intestine length required for sufficient nutrient uptake remains a topic of debate. All cases had a small bowel length of less than 100 cm within the first year of life, which is generally considered abnormal.^41^ Reference values of healthy neonates born 37–41 weeks of gestational age indicate a lower limit of 119.8 cm (90% confidence interval [CI] 68.9−161.5).^42^ During follow‐up, 60% of CSBS cases achieved enteral autonomy during a reported median follow‐up time of 15 months. Enteral autonomy is the capacity to sustain growth and development solely through enteral nutrition, without parenteral support. A risk factor for prolonged PN dependency is a small bowel length of <50 cm.^43^ In our analysis, the median [range] small bowel length was 50.0 [20–85] cm explaining that not all cases achieved enteral autonomy during their documented follow‐up. Other and we found no structural changes of the epithelium.^22^ However, unspecific changes of the epithelium, such as reductions in ileal goblet cells, inflammatory cell infiltration in the mucosal and diffused abnormal layers of the intestinal smooth muscle were described in some cases with CLMP mutation.^37^ In FLNA various changes were observed such as abnormal layering of the muscularis propria, argyrophilic neurons in the myenteric plexus and nerve fibers in the lamina propria.^19^, ^24^

Clinically challenging is the distinction of CSBS from other causes of congenital diarrhea as most CSBS cases manifested in the first week of life with (bilious) vomiting and diarrhea. Others and we performed a comprehensive work‐up for congenital diarrhea, which was unremarkable. Malrotation in radiological imaging may increase the suspicion of a CSBS and should prompt to determine the small bowel length.^27^, ^28^, ^31^ Small bowel length can be estimated by small bowel follow‐trough (SBFT); however, this may be challenging in cases of severe shortening, where complete passage of contrast cannot be adequately followed. In our case, computed tomography was performed, allowing precise measurement of bowel length using multiplanar reconstruction technology. Consequently, prioritizing radiological imaging such as SBFT or MRI/CT scan^44^ and genetic testing as the first line of diagnostic work‐up can help to avoid invasive diagnostic tests such as laparotomies. In addition, detection of malrotation by radiological imaging supports the suspicion of CSBS.^9^, ^19^, ^20^, ^22^, ^27^ No studies have specifically addressed a causal relationship between CSBS and malrotation. However, it is well‐documented that intestinal growth plays a crucial role in the formation of loops and the rotation of the intestine during embryological development.^45^

In most cases with genetic testing, autosomal recessive CLMP or X‐linked FLNA mutations were found. Not surprisingly, family history with consanguinity was found in 54% of reported CLMP cases. Various reports have described affected siblings in CLMP and FLNA.^2^, ^13^, ^15^, ^28^, ^30^ Despite identical underlying genetic mutations, phenotypes varied among some siblings in terms of comorbidities, complications, and the duration of PN before achieving enteral autonomy.

The CLMP gene, located on chromosome 11 (11q24.1), encodes a transmembrane protein that plays a significant role in intestinal development.^5^ Various homozygous and compound heterozygous loss‐of‐function mutations in CLMP have been identified in cases with CSBS without specific identified genotype–phenotype correlations.^2^ In our analysis, CLMP compared to FLNA and the other mutations exhibit the fewest comorbidities, which were typically confined to the gastrointestinal system. Despite several studies investigating the role of CLMP in intestinal development, the precise pathomechanism by which CLMP mutations contribute to the reduced intestinal length observed in CSBS patients remains unclear.^2^, ^46^, ^47^ Whether the recent study by Luissint et al., which showed that CLMP expression is reduced in human colorectal cancer samples, has a prognostic relevance for CSBS cases with a CLMP mutation, is not known.^47^ Reassuring, we found no CLMP case with a malignant transformation, however follow‐up times were short. It needs to be further explored, whether screening for colorectal cancer is indicated for these patients.

The mainstay of treatment for CSBS is conservative management, including PN to ensure adequate caloric and nutrient intake. Main complications of PN include catheter‐associated sepsis, which was the main reason for death in the analyzed cohort.^18^, ^27^, ^31^, ^34^ To date, no immunodeficiency associated with CSBS has been described. In addition, our patient developed a catheter‐associated sepsis without that we found evidence of an immunodeficiency. One achievement of the last decade is the establishment of home PN, which significantly decreased catheter‐associated infections in children with short gut.^48^ Intestinal growth‐promoting treatments such as glucagon‐like peptide‐2 (GLP‐2) and surgical options to promote enteral autonomy and avoid complications have not yet been investigated.

In contrast to CLMP, patients harboring FLNA mutations tend to experience a more severe phenotype with diverse comorbidities, including cardiovascular (patent ductus arteriosus and atrial septal defect) as well as neurological abnormalities (retro cerebellar cysts).^24^, ^28^ Furthermore, FLNA cases often display characteristic dysmorphic features, such as facial anomalies, irregular toe implants, and sacral dimples. The role of FLNA in intestinal development is still unclear. It encodes an acytoskeletal protein which regulates cell shape by cross‐linking actin filaments^4^ and thereby it modulates the cellular response to chemical and mechanical environmental triggers. FLNA is known to play an important role in vascular development and cardiac morphogenesis.^49^ FLNA follows an X‐linked inheritance and only one study mentioned the clinical phenotype of a mother who experience seizures during pregnancy and was diagnosed with periventricular nodular heterotopia, which is part of the clinical spectrum of FLNA.^28^ Unfortunately, no genetic testing was performed to confirm her carrier status.

In addition to CLMP and FLNA mutations, our analysis confirms a broader genetic heterogeneity of CSBS with additional mutations in DYNC2LI1 gene, HLX gene, FOXF1 gene and duplication of chromosome 16q24.1 involving FOXF1.^6^, ^7^, ^8^, ^9^, ^17^ The role of these genes or their respective mutations in the development of the intestine has not yet been clarified.

The main limitation of our study is the potential for publication bias and the unstructured reporting of cases with missing information. Not all variables we analyzed were reported in all cases, including the length of the small intestine, information on the duration of PN or the achievement of enteral autonomy. As the overall follow‐up time was relatively short, achievement of enteral autonomy is probably underestimated. A major limitation is the insufficient reporting of disease progression in the analyzed cases. Future case reports or series should strive to systematically collect and report longitudinal data.

## 5 CONCLUSION

In conclusion, the prognosis of CSBS seems to depend mainly on the affected gene and complications of PN. The rate of achieving enteral nutrition is high. Radiological imaging and genetic testing are the diagnostic tools of choice.

## CONFLICT OF INTEREST STATEMENT

The authors declare no conflicts of interest.

## Supporting information

Supporting file 1.

Supporting file 2.

Supporting file 3.

## References

[1] Hamilton J , Reilly BJ , Morecki R . Short small intestine associated with malrotation: a newly described congenital cause of intestinal malabsorption. Gastroenterology. 1969;56:124‐136. 5765427

[2] Van Der Werf CS , Wabbersen TD , Hsiao NH , et al. CLMP is required for intestinal development, and loss‐of‐function mutations cause congenital short‐bowel syndrome. Gastroenterology. 2012;142(3):453‐462.e3. 22155368 10.1053/j.gastro.2011.11.038

[3] van der Werf CS , Sribudiani Y , Verheij JB , et al. Congenital short bowel syndrome as the presenting symptom in male patients with FLNA mutations. Genet Med. 2013;15(4):310‐313. 23037936 10.1038/gim.2012.123

[4] Feng Y , Walsh CA . The many faces of filamin: a versatile molecular scaffold for cell motility and signalling. Nat Cell Biol. 2004;6(11):1034‐1038. 15516996 10.1038/ncb1104-1034

[5] Raschperger E , Engstrom U , Pettersson RF , Fuxe J . CLMP, a novel member of the CTX family and a new component of epithelial tight junctions. J Biol Chem. 2004;279(1):796‐804. 14573622 10.1074/jbc.M308249200

[6] Bryson LJ , Flynn DM , Sabharwal A , Ahmed SF , Kinning E . A child with congenital short gut associated with DYNC2LI1 ciliopathy. Clin Dysmorphol. 2021;30(1):66‐68. 32815859 10.1097/MCD.0000000000000341

[7] Farrell SA , Sodhi S , Marshall CR , et al. HLX is a candidate gene for a pattern of anomalies associated with congenital diaphragmatic hernia, short bowel, and asplenia. Am J Med Genet A. 2017;173(11):3070‐3074. 28898547 10.1002/ajmg.a.38354

[8] Stankiewicz P , Sen P , Bhatt SS , et al. Genomic and genic deletions of the FOX gene cluster on 16q24.1 and inactivating mutations of FOXF1 cause alveolar capillary dysplasia and other malformations. Am J Hum Genet. 2009;84(6):780‐791. 19500772 10.1016/j.ajhg.2009.05.005 PMC2694971

[9] Lavillaureix A , Foulon G , Launay E , et al. Antenatal finding of 16q24.1 duplication including FOXF1, revealing an autosomal dominant familial pathology with congenital short bowel, malrotation and renal abnormalities. Clin Res Hepatol Gastroenterol. 2021;45(5):101562. 33208297 10.1016/j.clinre.2020.10.007

[10] Hou JW , Wang TR . Amelia, dextrocardia, asplenia, and congenital short bowel in deleted ring chromosome 4. J Med Genet. 1996;33(10):879‐881. 8933346 10.1136/jmg.33.10.879 PMC1050772

[11] De Backer AI , Parizel PM , De Schepper A , Vaneerdeweg W . A patient with congenital short small bowel associated with malrotation. J Belge Radiol. 1997;80(2):71‐72. 9237417

[12] Negri E , Coletta R , Morabito A . Congenital short bowel syndrome: systematic review of a rare condition. J Pediatr Surg. 2020;55(9):1809‐1814. 32278545 10.1016/j.jpedsurg.2020.03.009

[13] Alves MM , Halim D , Maroofian R , et al. Genetic screening of congenital short bowel syndrome patients confirms CLMP as the major gene involved in the recessive form of this disorder. Eur J Hum Genet. 2016;24(11):1627‐1629. 27352967 10.1038/ejhg.2016.58 PMC5055811

[14] Chu SM , Luo CC , Chou YH , Yen JB . Congenital short bowel syndrome with malrotation. Chang Gung Med J. 2004;27(7):548‐550. 15508878

[15] Chuang YH , Fan WL , Chu YD , et al. Whole‐exome sequencing identified novel CLMP mutations in a family with congenital short bowel syndrome presenting differently in two probands. Front Genet. 2020;11:574943. 33384711 10.3389/fgene.2020.574943 PMC7770137

[16] De Greef E , Mahler T , Janssen A , Cuypers H , Veereman‐Wauters G . The influence of neocate in paediatric short bowel syndrome on PN weaning. J Nutr Metab. 2010;2010:297575. 20721339 10.1155/2010/297575 PMC2915748

[17] Dharmadhikari AV , Gambin T , Szafranski P , et al. Molecular and clinical analyses of 16q24.1 duplications involving FOXF1 identify an evolutionarily unstable large minisatellite. BMC Med Genet. 2014;15:128. 25472632 10.1186/s12881-014-0128-z PMC4411736

[18] Erez I , Reish O , Kovalivker M , et al. Congenital short‐bowel and malrotation: clinical presentation and outcome of six affected offspring in three related families. Eur J Pediatr Surg. 2001;11(5):331‐334. 11719873 10.1055/s-2001-18546

[19] Gargiulo A , Auricchio R , Barone MV , et al. Filamin A is mutated in X‐linked chronic idiopathic intestinal pseudo‐obstruction with central nervous system involvement. Am J Hum Genet. 2007;80(4):751‐758. 17357080 10.1086/513321 PMC1852717

[20] Geng L , Zhou L , Ding GJ , et al. Alternative technique to save ischemic bowel segment in management of neonatal short bowel syndrome: a case report. World J Clin Cases. 2019;7(20):3353‐3357. 31667191 10.12998/wjcc.v7.i20.3353 PMC6819291

[21] Gharesouran J , Esfahani BS , Valilou SF , et al. First report of congenital short bowel syndrome in an Iranian patient caused by a mutation in the CLMP gene. J Pediatr Genet. 2019;8(2):73‐80. 31061750 10.1055/s-0038-1675339 PMC6499612

[22] Gonnaud L , Alves MM , Cremillieux C , et al. Two new mutations of the CLMP gene identified in a newborn presenting congenital short‐bowel syndrome. Clin Res Hepatol Gastroenterol. 2016;40(6):e65‐e67. 27720179 10.1016/j.clinre.2015.12.018

[23] Hasosah M , Lemberg DA , Skarsgard E , Schreiber R . Congenital short bowel syndrome: a case report and review of the literature. Can J Gastroenterol. 2008;22(1):71‐74. 18209785 10.1155/2008/590143 PMC2659124

[24] Kapur RP , Robertson SP , Hannibal MC , et al. Diffuse abnormal layering of small intestinal smooth muscle is present in patients with FLNA mutations and x‐linked intestinal pseudo‐obstruction. Am J Surg Pathol. 2010;34(10):1528‐1543. 20871226 10.1097/PAS.0b013e3181f0ae47

[25] Martucciello G , Torre M , Pini Prato A , et al. Associated anomalies in intestinal neuronal dysplasia. J Pediatr Surg. 2002;37(2):219‐223. 11819202 10.1053/jpsu.2002.30258

[26] Naik D , Mahalik SK , Pati AB . Congenital short bowel syndrome with annular pancreas presenting as neonatal intestinal obstruction. Cureus. 2022;14(11):e31802. 36579202 10.7759/cureus.31802 PMC9788789

[27] Nhan VT , Hoang TV , Nhan PNH , Huynh QTV , Ban HT . Congenital short bowel syndrome: cases series in the same family and review of literature. Int J Surg Case Rep. 2024;123:110135. 39173431 10.1016/j.ijscr.2024.110135 PMC11388007

[28] Oegema R , Hulst JM , Theuns‐Valks SD , et al. Novel no‐stop FLNA mutation causes multi‐organ involvement in males. Am J Med Genet A. 2013;161A(9):2376‐2384. 23873601 10.1002/ajmg.a.36109

[29] Ordonez P , Sondheimer JM , Fidanza S , Wilkening G , Hoffenberg EJ . Long‐term outcome of a patient with congenital short bowel syndrome. J Pediatr Gastroenterol Nutr. 2006;42(5):576‐580. 16707984 10.1097/01.mpg.0000189360.84169.da

[30] Ou FF , Li MJ , Mei LB , Lin XZ , Wu YA . Congenital short‐bowel syndrome is associated with a novel deletion mutation in the CLMP gene: mutations in CLMP caused CSBS. Front Pediatr. 2021;9:778859. 35111702 10.3389/fped.2021.778859 PMC8802778

[31] Palle L , Reddy B . Case report: congenital short bowel syndrome. Indian J Radiol Imaging. 2010;20(3):227‐229. 21042453 10.4103/0971-3026.69366 PMC2963747

[32] Sabharwal G , Strouse PJ , Islam S , Zoubi N . Congenital short‐gut syndrome. Pediatr Radiol. 2004;34(5):424‐427. 14676985 10.1007/s00247-003-1087-2

[33] Sala D , Chomto S , Hill S . Long‐term outcomes of short bowel syndrome requiring long‐term/home intravenous nutrition compared in children with gastroschisis and those with volvulus. Transplant Proc. 2010;42(1):5‐8. 20172269 10.1016/j.transproceed.2009.12.033

[34] Scheida N , Wales PW , Krishnamurthy G , Chait PG , Amaral JG . Ectopic drainage of the common bile duct into the lesser curvature of the gastric antrum in a newborn with pyloric atresia, annular pancreas and congenital short bowel syndrome. Pediatr Radiol. 2009;39(1):66‐69. 18818913 10.1007/s00247-008-1008-5

[35] Shehata B , Chang T , Greene C , et al. Gastric heterotopia with extensive involvement of the small intestine associated with congenital short bowel syndrome and intestinal malrotation. Fetal Pediatr Pathol. 2011;30(1):60‐63. 21204668 10.3109/15513815.2010.505631

[36] Siva C , Brasington R , Totty W , Sotelo A , Atkinson J . Synovial lipomatosis (lipoma arborescens) affecting multiple joints in a patient with congenital short bowel syndrome. J Rheumatol. 2002;29(5):1088‐1092. 12022328

[37] Wang Y , Chen S , Yan W , et al. Congenital short‐bowel syndrome: clinical and genetic presentation in China. J Parenter Enteral Nutr. 2021;45(5):1009‐1015. 10.1002/jpen.1974 33464596

[38] Zain M , Abdelkader M , Azab A , Kotb M . Congenital short bowel syndrome: a rare cause of neonatal intestinal obstruction. J Int Med Res. 2020;48(9):300060520954726. 32951488 10.1177/0300060520954726 PMC7509730

[39] Zhang W , Wu Y , Pan C , et al. Ruptured giant omphalocele with congenital short small intestine: a case report. Front Nutr. 2024;11:1421033. 39091686 10.3389/fnut.2024.1421033 PMC11291450

[40] Struijs MC , Diamond IR , de Silva N , Wales PW . Establishing norms for intestinal length in children. J Pediatr Surg. 2009;44(5):933‐938. 19433173 10.1016/j.jpedsurg.2009.01.031

[41] Coletta R , Khalil BA , Morabito A . Short bowel syndrome in children: surgical and medical perspectives. Semin Pediatr Surg. 2014;23(5):291‐297. 25459014 10.1053/j.sempedsurg.2014.09.010

[42] Bardwell C , El Demellawy D , Oltean I , et al. Establishing normal ranges for fetal and neonatal small and large intestinal lengths: results from a prospective postmortem study. World J Pediatr Surg. 2022;5(3):e000397. 36475045 10.1136/wjps-2021-000397 PMC9717316

[43] Fallon EM , Mitchell PD , Nehra D , et al. Neonates with short bowel syndrome: an optimistic future for parenteral nutrition independence. JAMA Surg. 2014;149(7):663‐670. 24827450 10.1001/jamasurg.2013.4332

[44] Chacon MA , Wilson NA . The challenge of small intestine length measurement: a systematic review of imaging techniques. J Surg Res. 2023;290:71‐82. 37210758 10.1016/j.jss.2023.04.011 PMC10330168

[45] Kluth D , Jaeschke‐Melli S , Fiegel H . The embryology of gut rotation. Semin Pediatr Surg. 2003;12(4):275‐279. 14655167 10.1053/j.sempedsurg.2003.08.009

[46] Langhorst H , Juttner R , Groneberg D , et al. The IgCAM CLMP regulates expression of Connexin43 and Connexin45 in intestinal and ureteral smooth muscle contraction in mice. Dis Model Mech. 2018;11(2):dmm032128. 29361518 10.1242/dmm.032128 PMC5894946

[47] Luissint AC , Fan S , Nishio H , et al. CXADR‐like membrane protein regulates colonic epithelial cell proliferation and prevents tumor growth. Gastroenterology. 2024;166(1):103‐16.e9. 37716376 10.1053/j.gastro.2023.09.012

[48] Hill S , Ksiazyk J , Prell C , Tabbers M . Nutrition EEECwgopp ESPGHAN/ESPEN/ESPR/CSPEN guidelines on pediatric parenteral nutrition: home parenteral nutrition. Clin Nutr. 2018;37(6 Pt B):2401‐2408. 30098848 10.1016/j.clnu.2018.06.954

[49] Robertson SP . Filamin A: phenotypic diversity. Curr Opin Genet Dev. 2005;15(3):301‐307. 15917206 10.1016/j.gde.2005.04.001

