# Supplementary material for: Congenital short bowel syndrome: Clinical aspects by systematic review
Source: JPGN Rep. 2026 Aug 13:10.1002/jpr3.70221. Online ahead of print. doi: 10.1002/jpr3.70221 (PMC13470618; doi:10.1002/jpr3.70221)
Supplement: Supplementary file 1 [file JPR3-9999-0-s001.docx]

**Supplementary Table 1**: Overview enteral autonomy

|  | Enteral autonomy achieved | Enteral autonomy not achieved |
| --- | --- | --- |
| N | 24 | 16 |
| No. mentioned small bowel length, n | 19 | 12 |
| Small bowel length, cm, median [range] | 50 [20-75] | 43.5 [20–85] |
| Duration follow-up, months, median [IQR] | 17.5 [12.5] | 8 [23.6] |
| Genetic background | CLMP in 9, FLNA 4, other mutations 1, no genetics performed 10 | CLMP in 2, FLNA 3, other mutations 2, no genetics performed 9 |

IQR: interquartile range, CLMP: Coxsackie and Adenovirus receptor-like membrane protein, FLNA: Filamin A
