# Supplementary material for: Congenital short bowel syndrome: Clinical aspects by systematic review
Source: JPGN Rep. 2026 Aug 13:10.1002/jpr3.70221. Online ahead of print. doi: 10.1002/jpr3.70221 (PMC13470618; doi:10.1002/jpr3.70221)
Supplement: Supplementary file 2 [file JPR3-9999-0-s002.docx]

Publications included

(n = 32)

**Inclusion**

**Screening**

Abstracts excluded

(n = 1)

Full texts excluded:

No new patients’ data (n = 9)

Not congenital (n = 6)

Full text not English (n = 1)

Publications identified

(n = 45)

Abstracts screened

(n = 45)

Full text screening

(n = 44)

**Identification**

Full texts from reference lists of retrieved publications fulfilling inclusion criteria

(n = 4)

**Supplemental Figure 1**: Flow-diagram of inclusion process.
