# Supplementary material for: Congenital short bowel syndrome: Clinical aspects by systematic review
Source: JPGN Rep. 2026 Aug 13:10.1002/jpr3.70221. Online ahead of print. doi: 10.1002/jpr3.70221 (PMC13470618; doi:10.1002/jpr3.70221)
Supplement: Supplementary file 3 [file JPR3-9999-0-s003.docx]

**Supplementary Table 2**: Overview of diagnostics in our patient

| **Differential diagnosis** | **Diagnostic tool and result** |
| --- | --- |
| **Infection** | |
| Norovirus, adenovirus, rotavirus, enterovirus | Viral stool cultures negatives |
| Cytomegalovirus | Polymerase Chain Reaction test quantitative blood negative |
| **Food intolerance** | |
| Congenital sucrase-isomaltase deficiency | Unlikely, as persistent diarrhea under amino-based infant formula |
| «Food protein-induced enterocolitis syndrome» (FPIES) | Unlikely, as persistent diarrhea under amino-based infant formula |
| **Metabolic disorders** | |
| Aminoazidopathia, organoazidopathia | Tandem mass spectrometry of amino acids unremarkable |
| Fatty acid oxidation disorders | Tandem mass spectrometry of acylcarnitines unremarkable |
| **Immunodeficiency** | |
| «Severe combined Immunodeficiency» (SCID), Agammaglobulinemia | Neutrophil granulocyte count 2.6 G/l [1.5–7.5 G/l] normal  Lymphocyte count 12.18 G/l [3.0–12.5 G/l] normal |
|  | Immunoglobuline (Ig)  IgG 6.0 g/l [1.5–6.3 g/l] normal  IgA 0.11 g/l [<0.83 g/l] normal  IgM 0.32 g/l [<1.45 g/l] normal |
|  | Newborn screening (SCID and severe T-cell lymphopenia) negative |
| «Immune dysregulation, polyendocrinopathy, enteropathy, X-linked»- (IPEX-) syndrome, ADAM metallopeptidase domain 17- (ADAM17-) deficiency | Clinical no skin or hair abnormalities, no endocrine disorders |
| **Structural changes of enterocytes and transporter defects** | |
| Intestinal epithelial dysplasia (tufting enteropathy) | Hematoxylin and eosin staining unremarkable, no typical clustering of enterocytes (tufts) |
| Microvillus inclusion disease | No electron microscopy |
| Congenital chloride diarrhea  Congenital sodium diarrhea | Blood electrolytes unremarkable, no alkalosis  Stool electrolytes taken three times, but no analysis possible (twice stool too dry, once insufficient sample material). |
| **Others** | |
| Intestinal lymphangiectasia (primary/secondary) | IgG 6.0 g/l [1.5–6.3 g/l] normal |
|  | Lymphocyte count 12.18 G/l [3.0–12.5 G/l] normal |
|  | No evidence of dilated lymphatic vessels in histology |

Reference values are indicated with square brackets.
